# Effects of Training Body-Related Interpretations on Panic-Related Cognitions and Symptoms

**DOI:** 10.1007/s10608-023-10358-9

**Published:** 2023-02-09

**Authors:** Felix Würtz, Shari Steinman, Simon E. Blackwell, Frank H. Wilhelm, Andrea Reinecke, Dirk Adolph, Jürgen Margraf, Marcella L. Woud

**Affiliations:** 1 Mental Health Treatment and Research Center, Faculty of Psychology, Ruhr-University Bochum, Massenbergstraße 9-13, D-44787 Bochum, Germany; 2 Psychology Department, West Virginia University, Morgantown, USA; 3 Division of Clinical Psychology and Psychopathology, Department of Psychology, Paris- Lodron University Salzburg, Hellbrunner Straße 34, 5020 Salzburg, Austria; 4 Department of Psychiatry, University of Oxford, Oxford , UK; 5 Oxford Health NHS Foundation Trust, Oxford , UK

**Keywords:** Cognitive bias modification, Interpretation bias, Panic disorder, Symptom provocation, Experimental psychopathology

## Abstract

**Background:**

Interpretation biases (IBs) are central in panic disorder, and there is rich evidence showing that these are correlated with and predictive of panic-relevant symptomatology. However, experimental studies are needed to examine the potential causal effects of IBs, as predicted by cognitive models.

**Methods:**

Panic-related IBs were manipulated via a sentence-completion Cognitive Bias Modification-Interpretation (CBM-I) training. The sample included *N* = 112 healthy participants reporting moderate levels of fear of bodily sensations. Participants were randomly allocated to a positive, negative, or control CBM-I condition. To test the trainings’ effect on panic-relevant cognitive processing, IBs were assessed via proximal and distal measures. Symptom provocation tasks were applied to test transfer to panic-relevant symptomatology.

**Results:**

Results on the proximal measure showed that positive CBM-I led to more positive IBs compared to negative, and control training. Further, positive CBM-I led to more positive IBs on the distal measure as compared to negative CBM-I. However, there were no differential training effects on panic-related symptomatology triggered via the provocation tasks.

**Conclusion:**

The findings indicate a limited generalization of the effects of CBM-I on IBs and panic-related symptoms. Potential means to improve generalization, such as applying more nuanced measures and combining CBM-I with psychoeducation are discussed.

Cognitive models of panic disorder (PD; Clark 1986; Margraf & Schneider, 1990) postulate that negative interpretation biases (IBs), namely the tendency to interpret ambiguous, body-related information in a negative or catastrophizing manner, are central to the development and maintenance of the disorder. IBs in the context of PD typically relate to catastrophic misinterpretations of ambiguous bodily symptoms occurring in an arbitrary everyday-life situation, for example interpreting sudden palpitations as a sign of a heart-attack. Evidence for the central role of body-related IBs in the context of panic comes from several lines of research. For example, it has been shown that IBs are strongly associated with anxiety sensitivity (Olthuis et al., 2012; Teachman, 2005; Zahler et al., 2020), a concept describing the concern about experiencing symptoms of anxiety which in turn is predictive of the new onset of PD (Woud et al., 2014) and commonly applied as a subclinical panic analog (cf. Zahler et al., 2020). In clinical samples, Teachman et al. (2007) found that compared to healthy controls, patients with PD interpreted ambiguous scenarios describing bodily symptoms as more threatening (for similar results, see McNally & Foa 1987; Richards et al., 2001), and reported greater anxiety levels during a symptom provocation, with anxiety levels in turn being associated with the IBs. In another cross-sectional study Hermans et al. (2010) found that patients with PD showed more negative IBs concerning bodily symptoms compared to patients with other anxiety disorders and healthy controls. Besides this cross-sectional evidence, it has been shown that IBs were predictive of new onset of PD (Woud et al., 2014). Moreover, the reduction of IBs precedes symptom improvement during cognitive behavioral therapy for PD but not vice versa (Teachman et al., 2010). This provides further evidence for IBs not only being a correlate but also a risk factor of PD (for reviews on the association between IBs and anxiety disorders, see Hirsch et al., 2016; Ohst & Tuschen-Caffier, 2018). While these findings offer correlational evidence for the central role of IBs in panic, investigating the potential causal role of IBs is the next crucial and critical step to investigate predictions of cognitive models of PD. This step, in turn, requires manipulating IBs, i.e., reducing and inducing them, followed by examining the effects of such a manipulation on panic-related symptoms (Kraemer et al., 1997).

Techniques for the manipulation of IBs have been developed within the framework of Cognitive Bias Modification for Interpretations (CBM-I; Koster et al., 2009; Woud & Becker, 2014). CBM-I involves a range of computerized tasks during which participants see ambiguous, incomplete information (e.g., an open-ended sentence) that can only be completed according to participants’ allocated training condition, e.g., in a positive or negative manner. One example are sentence completion paradigms (cf. MacLeod & Mathews, 2012) during which participants are presented with disorder-related, ambiguous open-ended sentences (e.g., *A strange feeling in my stomach signals that I am very…*), and are instructed to resolve this ambiguity by finishing a subsequently presented word fragment (e.g., positive resolution: “h_ppy”, *happy*; negative resolution: “i_l” *ill*). Importantly, each word fragment is chosen such that it resolves the ambiguity in a training-congruent manner. To illustrate, in a training designed to induce more positive interpretations, positive and functional words are used for the word fragment, and the ambiguity is thus consistently resolved positively. To date, there are several studies showing positive effects of CBM-I on (analog) symptoms of various psychopathologies (see for example Hirsch et al., 2018; Woud et al., 2021; for a recent network meta-analysis, see Fodor et al., 2020), yet the literature in the context of PD is still in its infancy.

In one of the first studies, Steinman & Teachman (2010) tested the effects of a positive versus control CBM-I training on panic-related IBs, fear of bodily symptoms, and responses to a panic symptom provocation task in individuals scoring high on anxiety sensitivity. As expected, the positive training led to a more positive IB and reduced anxiety sensitivity, compared to the control conditions. However, at post-training, the groups did not differ in more panic-specific symptomatology, including fear of body symptoms or fear and avoidance during the symptom provocation task (for similar results, see Clerkin et al., 2015; MacDonald et al., 2013). Capron et al. (2017) combined CBM-I with a psychoeducation intervention and reported more promising results—they also successfully induced a more positive IB via a positive compared to a control condition receiving only psychoeducation and additionally found that positively trained participants reported both less fear during a symptom provocation task and lower scores on anxiety sensitivity. Finally, a recent study investigated the effects of a CBM-I training in a sample including diagnosed PD patients. Findings were promising such that a more positive IB was induced, and there was a significant panic symptom reduction from pre- to post-intervention (Beard et al., 2016). However, since the study did not include a control condition these results have to be interpreted with caution.

In summary, although there is initial evidence that CBM-I can induce positive IBs in the context of panic and that it affects panic-related symptomatology, there are at least two problems related to these previous studies. First, none of the earlier studies applied a design that included all relevant conditions to fully test the potential causal effect of IBs. That is, there is no study that investigated the effects of an induction versus reduction of panic-related IBs and compared these effects to a control condition. When aiming to test causality, however, such a design is needed to show that more negative IBs partly produce symptoms while their reduction leads to their decrease (cf. Kraemer et al., 1997). However, mixed findings preclude conclusions about training’s transfer effects at a symptom level. Adequately testing causality and obtaining stable behavioral transfer effects, however, are crucial from both a theoretical and applied perspective. Such knowledge will aid to refine and adapt models of psychopathology, allowing a more tailored development of potential interventions.

Accordingly, the aim of the present study was three-fold. The first aim was to investigate whether a positive versus negative CBM-I training would induce training-congruent IBs in comparison to a neutral control condition on an IB measure closely related to the CBM-I training, the Encoding Recognition Task (ERT). The second aim was to test the trainings’ far transfer effects, i.e., whether effects would generalize to another, more distal measure of IBs, namely to the Scrambled Sentences Task (SST). This was done to investigate whether CBM-I would affect IBs operationalized more broadly, and the SST was chosen in particular due to its consistent association with symptoms of psychopathology (Würtz et al., 2022). The third aim was to investigate transfer effects on analog panic-related symptomatology induced by a symptom provocation task, to shed light on the potentially causal relationship between IBs and self-reported reactions to a panic-related symptom provocation task symptoms. For the ERT, we hypothesized that participants in the positive CBM-I condition would show a more positive IB at post-training compared to the control and the negative condition, and the negative condition would show a more negative IB in comparison to the positive and the neutral condition. We further hypothesized that we would find the same pattern of results on a more distal task, the SST, indicating successful generalization. Finally, we hypothesized that participants in the positive condition would show a smaller increase in panic-related symptoms over the course of the symptom provocation tasks compared to the control and negative condition, whereas the negative condition would show a greater increase in such symptoms compared to the control and positive condition.

## Methods

### Pre-Registration and Open Material

Prior to the start of data collection, the study was pre-registered on AsPredicted (https://aspredicted.org/blind.php?x=X6L_2WQ**)** and all deviations from the registration are labelled as such. All materials (except for standardized questionnaires), including the analysis scripts and the anonymized dataset are openly available on the Open Science Framework (OSF; https://osf.io/92tfw/).

### Participants and Eligibility Screening

Healthy participants with moderate fear of bodily symptoms were recruited via social media and the online noticeboard of the Faculty of Psychology at Ruhr-University Bochum. Interested participants were sent a link to take part in an online screening. During the screening, participants’ demographic data was assessed including age, gender, educational level, and native language. Further, they were asked for current or past psychological disorders, suicidal ideation, and self-harming behavior. Also, their anxiety sensitivity was assessed using the Anxiety Sensitivity Index (ASI; Taylor et al., 2007) and their fear of bodily sensations via the Body Sensations Questionnaire (BSQ; Chambless et al., 1984). Finally, participants were asked for their regular physical activity via the movement- and sports activity questionnaire (BSA; Fuchs et al., 2015), as part of the cover story (see section *Cover story* for more details). After the screening, eligible participants were invited to the laboratory session via e-mail.

Eligibility criteria were the following: Age between 18 and 35 in order to recruit a homogenous sample, and a mean score between 1.5 and 2.7 on the Body Sensations Questionnaire (BSQ). The latter criterion was applied to allow both the induction and reduction of body related anxiety and associated IBs (for a similar procedure, see MacLeod et al., 2002) and based on the mean BSQ scores ± 1 SD of the healthy controls in an earlier study on panic disorder conducted at the same research department (Schneider & Schulte, 2007). Exclusion criteria were assessed via self-report and included: Current pregnancy, suicidal ideation, self-harming behavior, a severe cardiovascular or respiratory disease or malfunction, a current or past psychological disorder, and inability to perform physical exercise due to the potentially strenuous symptom provocation tasks. All study participants provided written informed consent and all study procedures were approved by the local ethics committee of the Faculty of Psychology at Ruhr-University Bochum (approval no. 506). Participants were reimbursed with either course credit or 30€.

### Cognitive Bias Modification-Interpretation (CBM-I) Training

The CBM-I training was an adapted sentence-completion paradigm (Mathews & Mackintosh, 2000), including training stimuli that were based on translated items of Steinman & Teachman (2010). In both valenced conditions (i.e., positive vs. negative), each training trial consisted of the presentation of an ambiguous, open-ended scenario targeting typical panic-related cognitions, followed by a word fragment that resolved the presented ambiguity in a training-congruent manner. Participants were instructed to complete the word fragment by typing in the first missing letter (e.g., *Sudden palpitations are a sign of …*, positive word fragment: *excit_ment*, resolved: *excitement*; negative word fragment: *hear_-attacks*, resolved: *heart-attacks*). Scenarios were taken from Steinman & Teachman (2010) together with positive resolutions while negative resolutions were newly created for this study. In the neutral control condition, solely panic-unrelated, ambiguous scenarios were presented, and these were resolved in 50% of the trials in a positive and negative manner, respectively (e.g., *A new pizza place has opened in town, the pizza tastes…*, word fragment: *go_d*, resolved: *good*). In total, there were 90 scenarios, divided over 10 blocks consisting of nine trials each. In each block of the positive and the negative condition, seven trials included panic-related scenarios and two trials were neutral fillers. For a third of the trials in both conditions, participants were asked to answer a short comprehension question about the last scenario presented to ensure ongoing engagement with the task. All computer programs were programmed in Inquisit 4 (Millisecond Software, 2014).

### Interpretation Bias Assessment

#### Encoding Recognition Task (ERT)

To assess whether the CBM-I training induced the expected IB, we used a near transfer measure closely related to the setup of the training, namely the Encoding Recognition Task (ERT; see e.g., Mathews & Mackintosh 2000; Würtz et al., 2021). The ERT consisted of two phases, an encoding- and a recognition phase. The encoding phase is similar to the CBM-I training such that participants were presented with ambiguous, open-ended panic-related scenarios, and instructed to complete these scenarios by typing in the first missing letter of a word fragment. However, in contrast to the CBM-I training, completing the word fragment did not resolve the ambiguity of the sentence. Further, each scenario was presented together with an introducing title. In the recognition phase, the titles of the scenarios were presented again, in randomized order, together with four novel, ambiguous sentences, related to the scenario associated with that title. Of these, one sentence resolved the ambiguity of the scenario in a positive (positive target) and one in a negative way (negative target). The two remaining sentences did not resolve the ambiguity yet had a generally positive (positive foil) or negative (negative foil) valence. Participants rated each of these sentences for their similarity in meaning to the corresponding scenario on a 4-point scale ranging from 1 (*not similar at all*) to 4 (*very similar*). The ERT consisted of 10 scenarios which were newly developed for this study to closely resemble scenarios presented in the CBM-I training.

The ERT bias score was calculated by subtracting the mean similarity ratings of the negative targets from the mean similarity ratings of the positive targets, resulting in a score ranging from − 4 to 4, with higher values indicating a more positive IB (for a similar procedure, see Würtz et al., 2021). There were two versions of the ERT presented before and after the CBM training in counterbalanced order (i.e., AB or BA). The reliability of the ERT bias score ranged from α [95%-CI] = 0.63 [0.49, 0.78] to α [95%-CI] = 0.86 [0.81, 0.91] across versions and applications.

#### Scrambled Sentences Task (SST)

To assess whether the effects of the CBM-I training would generalize to a more distal measure of IB, a computerized version of the SST was applied (Wenzlaff & Bates, 1998). It included panic-related stimuli that were developed by Zahler et al., (2020). During the SST, participants were presented with sets of six words and instructed to sort them into grammatically correct sentences by selecting five of them. In each trial, two words were target words, and the sentences were designed such that one of these target words had to be omitted to form a correct sentence. Depending on the omitted word, the selected words formed either a panic-related positive or negative interpretation of the sentence (e.g., Scrambled sentence: pressure chest in *dangerous* is *harmless*; Positive: Pressure in the chest is harmless, Negative: Pressure in the chest is dangerous).

The SST started with five neutral practice trials followed by 20 test trials. To reduce the amount of active evaluation during the task, a cognitive load was applied, i.e., prior to the task participants were instructed to keep a 6-digit number in mind and to report it at the end of the task. Additionally, each trial had a time limit of 12s. In total, 67.86% of participants remembered the 6-digit number correctly. 12.95% of the trials had to be excluded (total number of trials: 290) due to errors (grammatically incorrect sentences or fewer than 5 words selected within the time limit).

An SST bias score was calculated by dividing the number of negatively formed sentences by the total number of correctly formed sentences, resulting in an SST bias score ranging from 0 to 1, with higher values indicating a more negative IB. The split-half reliability of the SST was Split-half [95%-CI] = 0.71 [0.58, 0.81] (for a review and meta-analysis about the SST’s psychometric properties, see Würtz et al., 2022).

#### Symptom Provocation Tasks and Symptom Questionnaire

To investigate the effects of the CBM-I training on panic-related symptomatology, participants completed different symptom provocations tasks. These included physical exercises provoking symptoms typically described during panic attacks, such as palpitations or shortness of breath. There were two sets of symptom provocation tasks, each including three sub-tasks. The two sets were carried out before and after the CBM training, respectively. During the first set, participants were instructed to breathe through a narrow straw, to run on the spot, and to spin on a chair. During the second set, participants were instructed to blow out an imaginary candle as often as possible, to do jumping jacks, and to put a finger on a bottle on the floor and run around the bottle as fast as possible, head down while focusing on their finger that was placed on the bottle’s top. Each individual task lasted one minute (for similar tasks, see for example Steinman & Teachman 2010) and participants were informed that they could stop the task at any time.

The tasks’ effect was assessed using a self-made symptom questionnaire asking participants to rate the current strength of each of the 13 DSM-5 panic symptoms (e.g., palpitations, trembling, dizziness). The questionnaire’s last item asked about participants’ present level of general anxiety. All items had to be rated on an 11-point scale ranging from 0 (*not present*) to 10 (*extreme*). This questionnaire was applied four times, i.e., pre- and post a first and a second symptom provocation. The reliability of the symptom assessments ranged from α [95%-CI] = 0.85 [0.81, 0.89] to α [95%-CI] = 0.91 [0.88, 0.93] across questionnaire applications. No participant terminated the task before the time elapsed.

### Cover Story

To provide participants with a study narrative and to disguise the link between the CBM-I training and the symptom provocation tasks, the study was presented with a cover story. Specifically, it was explained that two distinct studies were conducted but combined, one focusing on general fitness and one on body-related cognitions. Hence, the screening included health-related questionnaires (see section *Participants and eligibility screening* for more information) and the symptom provocation tasks were introduced as being part of the study examining general fitness. During the first symptom provocation task, participants were equipped with a mobile heart rate monitor and told that they would receive feedback on their heart rate afterwards. This feedback, however, was standardized such that all participants were told that the heart rate curves seemed to show anomalies and that they should contact the study supervisor or their general practitioner afterwards. The aim of this bogus feedback was to activate awareness of bodily sensations and to induce a negative cognitive style concerning bodily symptoms to make the CBM-I training more relevant for participants.

### Feedback Questionnaire

At the end of the testing session, participants were asked to provide feedback about the study. Specifically, they were asked to rate (a) how strenuous the first symptom provocation was, (b) how exhausting the second symptom provocation was, and (c) how anxious they were due to the feedback after the first symptom provocation. All questions were answered using an 11-point scale ranging from 0 (*not at all*) to 10 (*extremely*). Finally, participants were asked to describe what they thought to be the study’s purpose was using an open-ended format.

### Baseline and Screening Questionnaires

To allow for a more fine-grained sample description and to support the cover story, participants answered the Depression Anxiety Stress Scale – 21 (DASS-21; Lovibond & Lovibond 1995), the Agoraphobic Cognitions Inventory (ACQ; Chambless et al., 1984) the state and trait version of the State-Trait Anxiety Inventory (STAI-S/T; Laux et al., 1981; Spielberger et al., 1983), and an assessment of the participants’ physical fitness via the Physical Fitness Questionnaire (FFB-Mot; Bös et al., 2002) as well as an activity questionnaire (BSA; Fuchs et al., 2015).

### Randomization

Participants were randomly allocated to one of the three CBM-I conditions via a randomization sequence generated by a researcher not involved in testing (SEB) using a true random number generator (www.random.org). To ensure that groups were balanced at baseline, randomization was stratified for gender (male vs. female), age (< 26.50 vs. ≥ 26.50), and mean BSQ score (< 2.10 vs. ≥ 2.10). To find out a participant’s allocation, the testing researcher entered a code into a Qualtrics survey, which then displayed the allocated condition. Hence, participant allocation was concealed until the point of randomization.

### Procedure

**Fig. 1:**
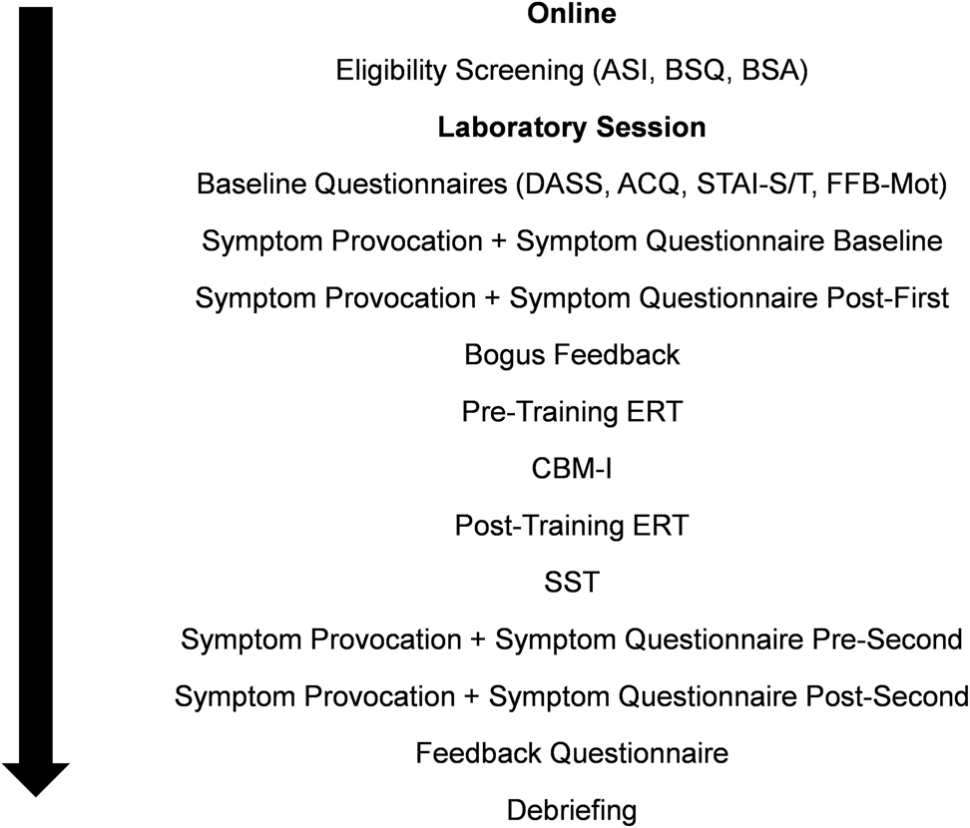
Graphical illustration of the study procedure

After completing the online screening (ASI, BSQ, BSA), eligible participants were invited for the laboratory part of the study. First, participants provided written informed consent and then filled in the baseline (DASS, ACQ, STAI-S/T, FFB-Mot) and symptom questionnaires. After that, participants did the first set of symptom provocation tasks, followed by the symptom questionnaire and the bogus feedback. For the latter, the experimenter left the room for several minutes to pick up the pulse curve results, which was then presented to the participants together with the bogus feedback. Next, participants completed the pre-training ERT and were then allocated to one of the three CBM-I conditions. After completing CBM-I, participants completed the post-training ERT and the SST. Then, participants completed the symptom questionnaire again, did the second set of symptom provocation tasks, followed by the final symptom questionnaire and the feedback questionnaire. Finally, participants allocated to the negative condition did the positive training to reduce the potential effects of the negative induction. Afterwards participants were debriefed and received their incentive for participation. For an overview of the study design, see Fig. 1.

### Statistical Analysis

#### Effects of CBM-I on IB Measures

Effects of the CBM-I training on the ERT and the SST bias score were investigated using one-way (Condition: Positive, Negative, Control) analysis of variances (ANOVAs) with the ERT bias score at post-training and the SST bias score as dependent variables, respectively. For the ERT analysis, the ERT at pre-training was entered as a covariate to control for pre-training scores (not collected for the SST). All significant effects were followed up using post-hoc pairwise comparisons with Bonferroni correction. In both analyses, we expected to find significant main effects of Condition, with more positive IBs (higher ERT and lower SST bias scores) in the positive condition compared to the control and negative condition, and vice versa for the negative condition.

#### Effects of CBM-I on Symptom Provocation Tasks 

Effects of the symptom provocation tasks were investigated using a 3 × 4 (Condition: Positive, Negative, Control; Time: Baseline, Post-First-Provocation, Pre-Second-Provocation, Post-Second-Provocation) linear mixed model with maximum likelihood estimation and a random intercept for individual participants. We expected to find a significant increase in panic symptoms from Baseline to Post-First-Provocation indicated by a significant main effect of Time, irrespective of the condition, indicated by a non-significant Time x Condition interaction. Concerning symptom levels at Pre-second provocation, we had no specific hypotheses regarding group differences or the comparison to Baseline. Finally, for the comparison between Baseline and Post-Second-Provocation, we expected a significant increase in panic-related symptoms, indicated by a significant main effect of Time. However, our main interest was on the Time x Condition interactions, such that we expected the increase in symptoms would be weaker in the positive compared to the control and to the negative condition, and vice versa for the negative condition. Analyses of the symptom provocation tasks differed from the pre-registered analyses such that we decided to run a linear mixed model including the scores of all four time points instead of running a repeated measures ANOVA including change scores. By doing so, we can account better for interindividual differences in the susceptibility to the effects of the symptom provocation tasks. Further, we were able to model potential differences in anticipatory anxiety which would be concealed by the aggregation of the symptom provocations into change scores. We considered this to be a more sensitive approach as Beard et al., (2016) found a significant effect of CBM-I on anticipatory but not on reactive anxiety.

#### Exploratory Analyses 

Via exploratory analyses we aimed to further investigate our pattern of results. Specifically, we calculated Spearman’s rho correlations with bootstrapped 95% confidence intervals (5,000 iterations) between the ERT at pre-training, the BSQ, and the symptom questionnaire at Baseline and post-First provocation to investigate the interrelation of IB, self-reported fear of body symptoms and provoked panic-related symptoms prior to the CBM manipulation. Spearman’s rho was chosen over planned Pearson correlations due to a skewed non-normal distribution of the symptom provocation data. Additionally, we calculated Spearman’s rho correlations between the ERT at post-training, the SST, and the symptom questionnaire before and after the provocation tasks at pre- and post-Second provocation to investigate whether the induced IB would be associated with provoked panic symptoms. In a second set of exploratory analyses, we repeated the analysis for the symptom provocation tasks using only the one-item anxiety ratings as dependent variables, to investigate whether the provocation tasks induced anxiety and whether this differed between groups.

We also compared the three Conditions on their answers on the feedback questionnaire using a one-way (Condition: Positive, Negative, Control) ANOVA to investigate whether the groups differed in how exhausting they found each symptom provocation task and how anxious they were due to the false feedback.

Finally, we used a Chi-Square test to compare the number of participants in each Condition who correctly guessed the study’s purpose. Participants’ open-ended answers were coded by two independent researchers and disagreement was resolved through discussion. Specifically, we classified answers as being indicative of awareness of the study purpose if they guessed that the computer program was a manipulation of the attitude towards, appraisal, or interpretations of body symptoms, and/or that the pulse curve from the heart rate monitor was fake.

#### Packages and Settings

All analyses were run in RStudio version 2022.7.1.554 (RStudio Team, 2022) using R version 4.0.0 (R Core Team, 2021). AN(C)OVAs were calculated using the package ‘afex’ (Singmann et al., 2020) with Type III sums of squares and using generalized η² (η²_G_) as effect sizes. Confidence intervals for η²_G_ were calculated using the package ‘effectsize’ (Ben-Shachar et al., 2020). Linear mixed models were computed using the package ‘nlme’ (Pinheiro et al., 2020) with dummy coding of the between- and within subject factors and the control condition before the first symptom provocation task as the reference category. Effect sizes for linear mixed models as well as all post-hoc pairwise comparisons were computed using the package ‘emmeans’ (Lenth, 2020) using the pooled SD as the denominator for effect sizes. Spearman correlations for the exploratory analyses were calculated using the packages ‘stats’ (R Core Team, 2021) and ‘rcompanion’ (Mangiafico, 2021) with 5,000 iterations for bootstrapped confidence intervals.

## Results

### Sample Characteristics

In total, 113 participants were recruited. After data collection, however, one participant (allocated to the control condition) had to be excluded due to a self-reported current depressive episode. The final sample consisted of *N* = 112 participants (75% female, *M*_Age_ = 23.77, *SD*_Age_ = 3.48, range = 18–34). The final sample size differed from the pre-registered planned sample size of *N* = 120, as, due to Covid-19 restrictions, the study could not be continued, and due to the relatively small deviation from the planned sample size, the recruitment was terminated. The conditions did not differ in their baseline characteristics. For a summary, see Table 1.

**Table 1:** Sample characteristics

Measure M (SD), range	CBM-I Negative *n* = 38	CBM-I Positive *n* = 38	CBM-I Control *n* = 36	Reliability α [95%-CI]	Statistics*
Age	23.74 (3.29), 19–32	23.87 (3.57), 18–34	23.69 (3.68), 19–34	–	*F*(2, 109) = 0.02, *p* = .975
Gender (F/M)	30/8	28/10	26/10	–	χ²(2) = 0.50, *p* = .779
ASI-3	13.45 (5.61), 3–27	12.89 (5.97), 2–26	12.92 (5.43), 4–25	0.73 [0.66, 0.80]	*F*(2, 109) = 0.12, *p* = .891
ACQ	20.82 (4.32), 15–34	19.29 (3.83), 15–31	21.13 (4.63), 16–33	0.73 [0.66, 0.79]	*F*(2, 109) = 2.00, *p* = .140
BSQ	35.76 (6.84), 26–46	34.53 (6.12), 26–45	35.50 (5.81), 26–46	0.60 [0.48, 0.71]	*F*(2, 109) = 0.41, *p* = .666
DASS-21-Total	20.82 (11.86), 4–56	16.77 (16.08), 0–66	19.41 (14.49), 2–58	0.87 [0.83, 0.90]	*F*(2, 109) = 0.79, *p* = .456
DASS-21-Depression	5.84 (5.45), 0–26	5.42 (7.19), 0–30	5.89 (5.83), 0–24	0.84 [0.80, 0.89]	*F*(2, 109) = 0.06, *p* = .937
DASS-21-Anxiety	4.16 (3.67), 0–14	3.16 (3.37), 0–12	4.50 (5.00), 0–18	0.54 [0.41, 0.67]	*F*(2, 109) = 1.10, *p* = .336
DASS-21-Stress	10.84 (6.83), 0–30	8.23 (8.45), 0–36	9.03 (7.61), 0–30	0.85 [0.81, 0.90]	*F*(2, 109) = 1.16, *p* = .317
STAI-S	35.63 (7.62), 22–52	33.71 (5.95), 20–48	35.79 (7.68), 22–50	0.87 [0.84, 0.91]	*F*(2, 109) = 0.99, *p* = .375
STAI-T	39.84 (8.03), 22–62	36.84 (8.12), 21–56	38.81 (10.30), 24–62	0.89 [0.86, 0.92]	*F*(2, 109) = 1.12, *p* = .329

*CBM-I * Cognitive Bias Modification-Interpretations, *ASI-3 * Anxiety Sensitivity Index – 3, *ACQ * Agoraphobic Cognitions Questionnaire, *BSQ* Body Sensations Questionnaire, *DASS-21 * Depression, Anxiety, Stress Scale – 21, *STAI-S/T*  State Trait Anxiety Inventory – State/Trait version. *Groups were compared using 3 × 1 (Condition: Negative, Positive, Control) ANOVAs for continuous variables and a χ²-test for gender

### Effects of CBM-I on IB Measures

#### ERT

**Fig. 2:**
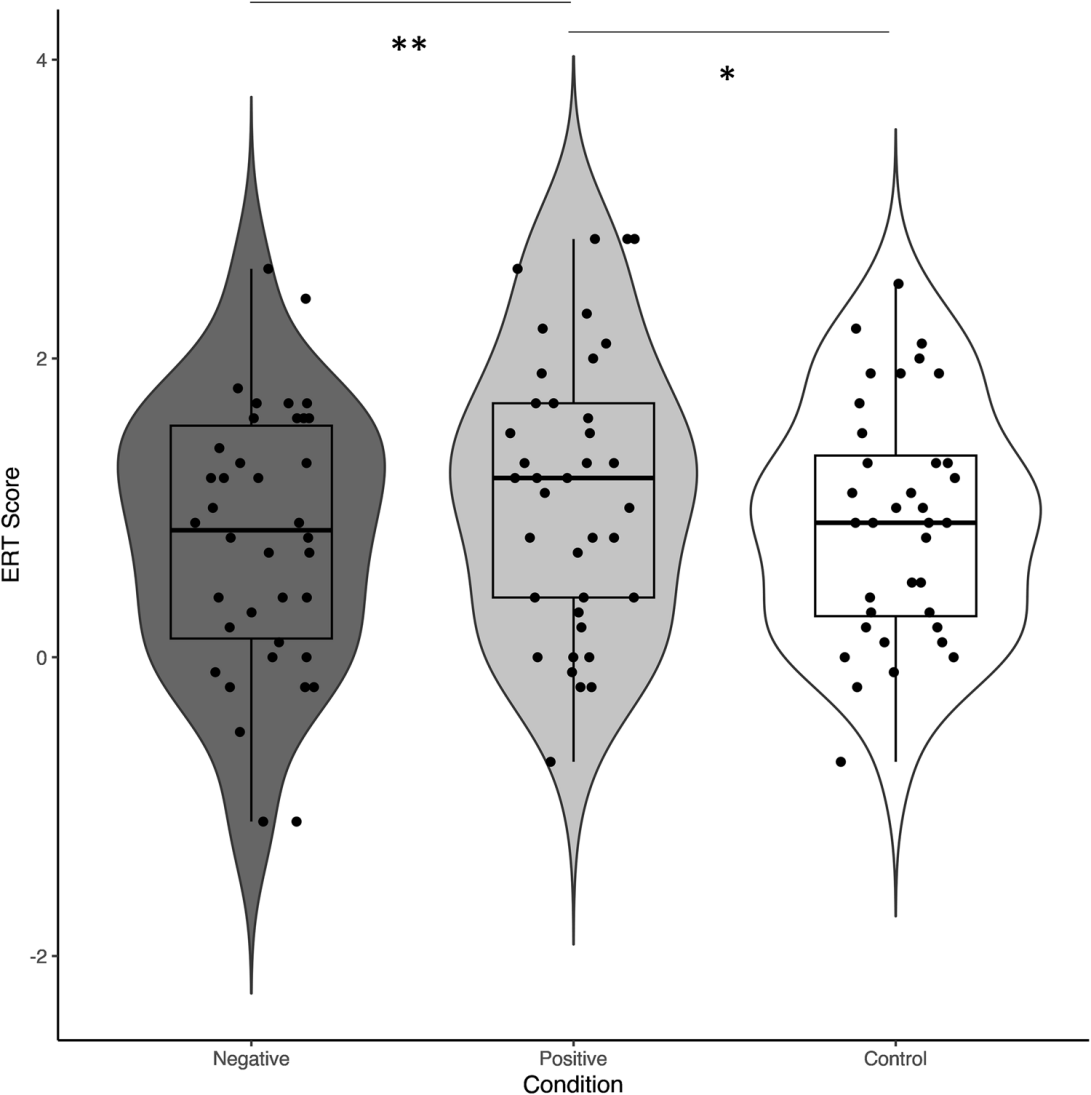
* *p* < .05, ** *p* < .01. Bias scores on the Encoding Recognition Task at post-training in each of the three CBM-I conditions

As expected, we found a significant main effect of Condition on the post-training ERT bias score, *F*(2, 108) = 6.21, *p* = .003, η_G_² [95%-CI] = 0.06 [0.00, 0.16]. Planned post-hoc comparisons revealed that this main effect was qualified by a significantly higher ERT bias score in the positive compared to the control condition, indicating a more positive IB at post-training for those trained positively, *t*(74) = 2.61, *p* = .033, *d* [95%-CI] = 0.48 [0.11, 0.86]. A similar result was found when comparing the positive versus negative condition, *t*(75) = 3.36, *p* = .004, *d* [95%-CI] = 0.58 [0.23, 0.94]. However, there was no significant difference between the negative and the control condition, *t*(74) = 0.71, *p* > .99, *d* [95%-CI] = 0.13 [− 0.24, 0.51]. For descriptive statistics of the outcome data, see Table 2 and for a graphical depiction, see Fig. 2.

**Table 2:** Descriptive statistics of variables of interest

Measure M (SD)	CBM-I Negative	CBM-I Positive	CBM-I Control
ERT-bias score pre-training	0.61 (0.73)	0.34 (0.76)	0.62 (0.70)
ERT-bias score post-training; EMM (SE)	0.73 (0.11)	1.26 (0.11)	0.84 (0.11)
SST score post-training	0.30 (0.18)	0.21 (0.13)	0.27 (0.16)
Symptom provocation Baseline	7.97 (17.2)	4.87 (5.83)	6.19 (8.84)
Symptom provocation Post-First	21.62 (17.85)	22.00 (14.98)	19.08 (10.86)
Symptom provocation Pre-Second	7.50 (15.73)	5.42 (5.66)	4.06 (3.44)
Symptom provocation Post-Second	23.39 (20.99)	22.61 (14.50)	20.08 (8.63)
Symptom provocation Baseline – anxiety	0.47 (0.86)	0.34 (0.88)	0.36 (0.99)
Symptom provocation Pre-First – anxiety	0.24 (0.91)	0.58 (1.76)	0.19 (0.67)
Symptom provocation Pre-Second – anxiety	0.29 (0.90)	0.24 (0.79)	0.17 (0.51)
Symptom provocation Post-Second – anxiety	0.55 (1.83)	0.39 (0.97)	0.06 (0.23)
Feedback – Exhaustion provocation 1	3.47 (2.27)	4.39 (2.28)	4.03 (2.36)
Feedback – Exhaustion provocation 2	4.63 (2.54)	4.87 (2.45)	4.31 (2.03)
Feedback – Anxiety feedback provocation 1	2.53 (2.59)	3.79 (2.84)	3.83 (3.05)
Awareness of study purpose (Yes/No)	5/33	9/29	4/32

For the ERT-bias score at post-training an estimated marginal mean (EMM) and the relevant standard error (SE) is presented to account for the ANCOVA which was used to control for the pre-training ERT-bias score. *ERT* Encoding Recognition Task. *SST * Scrambled Sentences Task. Higher scores on the ERT indicate a more positive interpretation bias, whereas higher scores on the SST indicate a more negative interpretation bias

#### SST

**Fig. 3:**
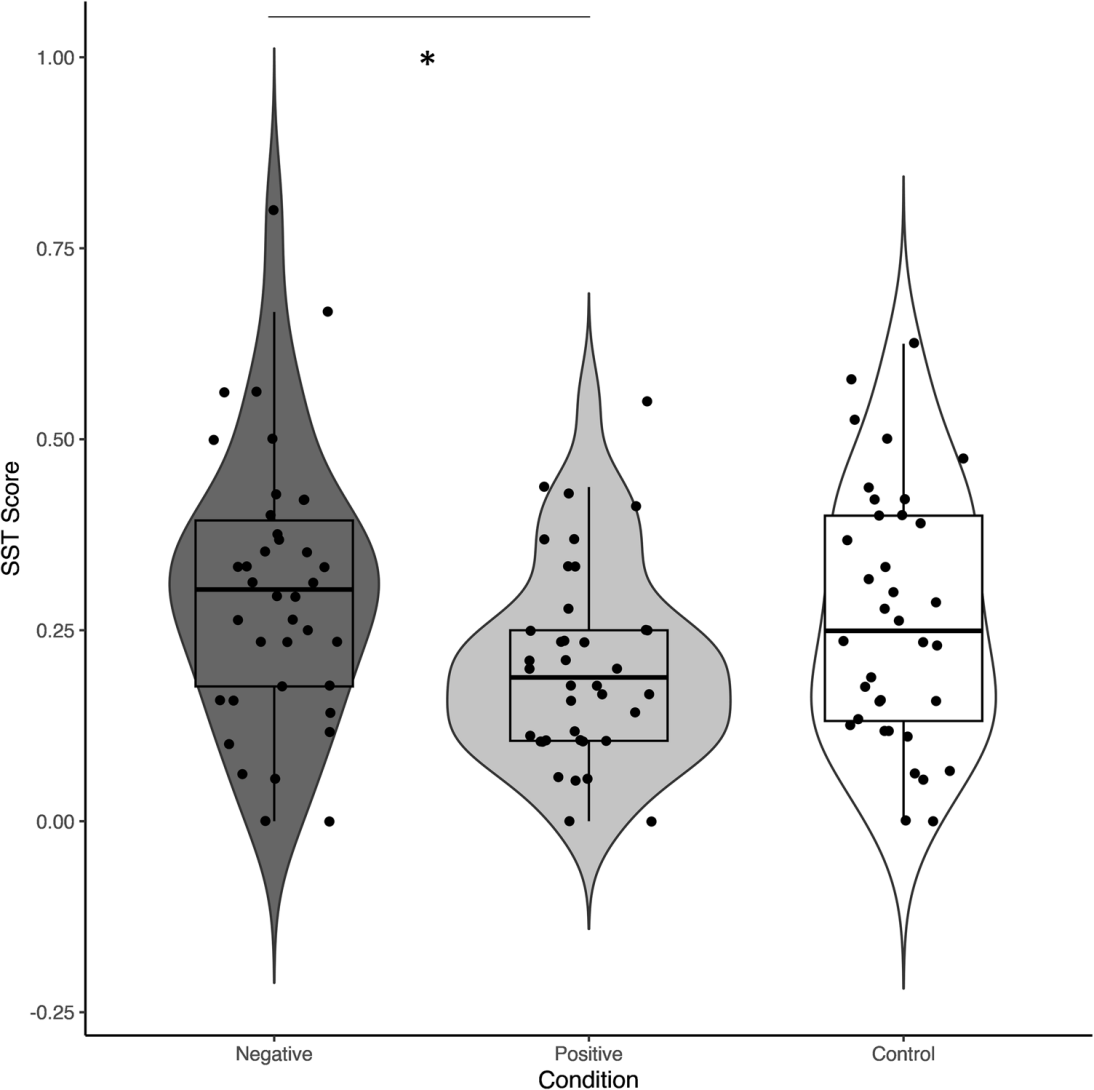
* *p* < .05. Bias scores on the Scrambled Sentences Task in each of the three CBM-I conditions

There was a significant main effect of Condition on the SST score, *F*(2, 109) = 3.79, *p* = .026, η_G_² [95%-CI] = 0.07 [0.00, 0.16]. Planned post-hoc comparisons revealed a significantly lower SST score in the positive compared to the negative condition, indicating a weaker negative IB for those trained positively, *t*(75) = 2.72, *p* = .024, *d* [95%-CI] = 0.64 [0.17, 1.12]. However, there were no significant differences for the remaining pairwise comparisons, *t*(75) < 1.71, *p* > .238, *d* < 0.22 indicating that neither the positive, nor the negative condition differed significantly from the control condition. For a graphical depiction, see Fig. 3.

#### Effects of CBM-I on Symptom Provocation Tasks

As expected, we found a significant main effect of Time comparing Post-First-Provocation to Baseline, *t*(327) = 6.73, *p* < .001, *d* [95%-CI] = 1.09 [0.87, 1.31], and none of the Time x Condition interactions were significant, *t*(327) < 1.59, *p* > .113. In sum, this indicates a significant and comparable increase in panic-related symptoms after the first symptom provocation task across all three conditions. Further, when comparing Pre-Second-Provocation to Baseline, there was neither a significant main effect of Time, *t*(327) = 1.12, *p* = .265, *d* [95%-CI] = 0.05 [-0.11, 0.21] nor significant Time x Condition interactions, *t*(327) < 1.01, *p* > .314, indicating that the level of panic symptoms across the three conditions did not differ from baseline to Pre-Second symptom provocation task.

**Fig. 4:**
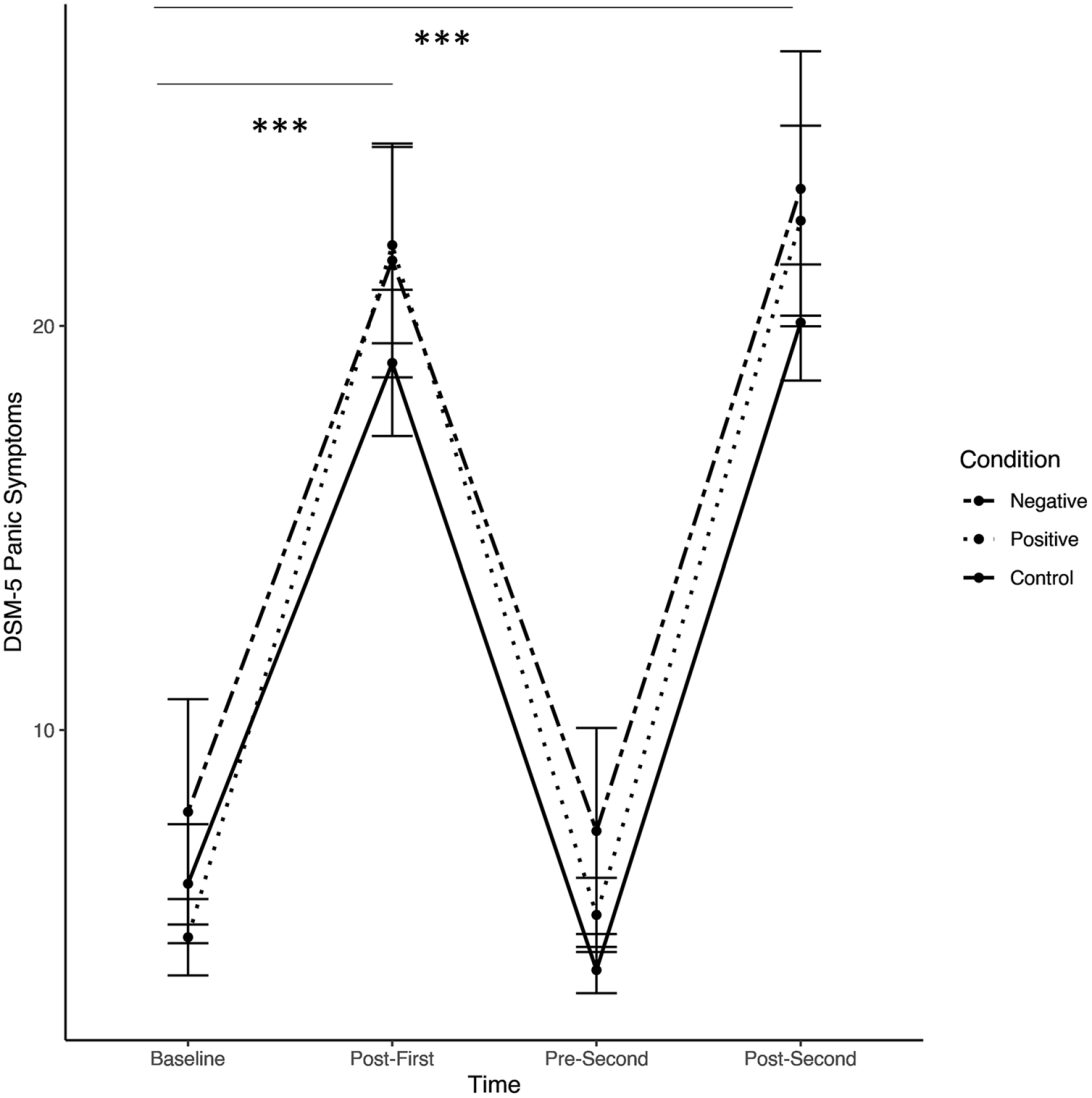
*** *p* < .001. Scores on the symptom questionnaire pre- and post each symptom provocation. Error bars represent standard errors

The comparison of Post-Second-Provocation to Baseline revealed a significant main effect of Time, *t*(327) = 7.25, *p* < .001, *d* [95%-CI] = 1.18 [0.94, 1.41], indicating a significant increase in panic-related symptoms across all three conditions. However, contrary to our expectations, the effect of main interest, the Time x Condition interaction was non-significant, *t*(327) < 1.44, *p* > .151. This suggests there was not a smaller increase in panic-related symptoms over the course of the symptom provocation tasks in the positive compared to the negative and control conditions, and not a larger increase in the negative compared to the positive and control condition. For a graphical depiction, see Fig. 4.

### Exploratory Analyses

#### Exploratory Correlations

Across the whole sample, the pre-training ERT was significantly negatively associated with the panic-related symptoms after the first symptom provocation task, indicating that participants with a more positive IB showed lower panic-related symptoms after the first symptom provocation. However, the remaining correlations at pre-training were non-significant, indicating that the level of self-reported fear of bodily symptoms (BSQ) was neither associated with the IB measured via the ERT, nor with body-related symptoms during the first provocation. For the post-training measures, the SST and the ERT were significantly and negatively correlated, indicating that a more positive IB on the ERT was associated with a less negative IB measured via the SST. However, the SST but not the ERT was significantly and positively correlated with symptoms before and after the second symptom provocation, showing that a more negative IB on the SST but not on the ERT was associated with more panic-related symptoms before and after the second provocation. For details, see Table 3.

**Table 3:** Exploratory correlations between variables of interest at pre- and post- training

Measure – pre-training	ERT-bias score pre-training	BSQ total score
ERT-bias score pre-training	–	
BSQ total score	.08 [−.11, .26]	–
Symptom provocation Baseline	.01 [−.18, .19]	.13 [−.06, .31]
Symptom provocation Post−First	−.19* [−.36, .00]	.12 [−.07, .30]

Measure – post-training	ERT-bias score post-training	SST score
ERT-bias score post-training	−	−
SST score	−.48*** [−.62, −.32]	−
Symptom provocation Pre-Second	−.10 [−.28, .09]	.28** [.11, .44]
Symptom provocation Post-Second	−.11 [−.29, .07]	.21* [.02, .39]

*p < .05, **p < .01, ***p < .001. Spearman’s rho correlation coefficients with bootstrapped 95%-CIs are presented

#### Anxiety During Symptom Provocation Tasks

To further specify our pattern of results, we examined the (differential) changes in anxiety during the symptom provocation tasks across the three conditions. There were no significant main effects or Time x Condition interactions, *t*(327) < 1.66, *p* > .098, indicating that anxiety ratings at no timepoint significantly differed from Baseline, irrespective of the allocated condition, and thus no anxiety was induced.

#### Feedback Questionnaire

Analyzing answers on the feedback questionnaire revealed that there was no difference between the conditions in how exhausting participants found the first or second symptom provocation task, *F*(2, 109) < 1.54, *p* > .219, η_G_² < 0.028, or how anxious they were due to the feedback after the first provocation task, *F*(2, 109) = 2.59, *p* = .079, η_G_² [95%-CI] = 0.045 [0.00, 0.13]. Investigating the open-ended feedback, 18 participants (16.07%) were (partly) aware of the study purpose or of the fact that the feedback after the first provocation was bogus, yet the conditions did not differ in whether participants were aware of the study purpose or not, χ^2^(2) = 2.53, *p* = .282. Repeating all analyses excluding participants who (partly) guessed the study purpose led to the same pattern of results.

## Discussion

The aim of the present study was to investigate whether a positive versus a negative panic-related CBM-I training would induce training-congruent IBs in comparison to a neutral control condition. The second aim was to test whether these effects would generalize to another, far transfer measure of IBs, namely the SST. The third aim was to investigate whether the positive and negative CBM-I training would have corresponding effects on participants’ reactions to various panic-related symptom provocation tasks, and thus whether panic-related IBs might be causally involved in the exacerbation of symptoms.

With regard to our first aim, our results were mixed in that we found a successful induction of a positive IB on the ERT in comparison to the negative and the control condition. However, the induction of a negative IB was only partly successful as the negative condition did not significantly differ from the control condition. Concerning our second aim, results indicated only a limited generalization effect on another measure of IBs, the SST. Here, we found a more positive IB in the positive than in the negative condition, yet neither the positive nor the negative condition differed significantly from the control condition. Finally, regarding our third aim, we found no effects of CBM-I on the symptom provocation tasks as indicated by no significant group differences regarding the increase in panic-related symptoms in the second symptom provocation compared to baseline.

Results concerning our first aim are generally in line with previous research on the effects of CBM-I in manipulating interpretations of bodily sensations in the context of panic (e.g., Capron et al., 2017; Steinman & Teachman, 2010). Comparable to previous work, we found that the positive condition successfully induced a more positive IB on the ERT compared to a neutral control and negative condition, whereby the latter comparison extends previous findings. However, as the negative condition did not differ from the neutral control condition, our aim of inducing a negative IB was not met. Hence, the hypotheses concerning the potentially causal role of IBs as suggested by cognitive models of panic could only be partly investigated. One reason for this finding could be that the strongly negative interpretations (e.g., sudden palpitations as a sign of a heart attack) were too extreme to be perceived as plausible in our healthy sample, and thus had no impact on participants’ interpretational style. Supporting this hypothesis, participants reported only little anxiety in response to the bogus feedback concerning their heart rate, questioning whether participants were actually susceptible to the induction of negative cognitions concerning their body symptoms.

Regarding the second aim of our study, our findings indicate a limited generalization of panic-related CBM-I effects to a more distal measure of IBs, the SST, which mirrors the findings by Steinman & Teachman (2010). The most parsimonious explanation for these findings relates to the small to medium effect sizes found for the post-training group comparisons on the ERT (see also Salemink et al., 2022). Since the ERT is closely matched with the CBM-I training, i.e., its stimuli and operationalization, one would expect medium-to-large effects on this measure (for a recent meta-analysis, see Martinelli et al., 2022). However, since we did not find such effects, it seems unlikely to then find large effects on the SST, a task that is conceptually even further away from the training. A second potential explanation for our mixed results is that we used a heterogeneous set of stimuli for both the training and the IBs’ assessment (i.e., ERT and SST). However, not all stimuli and related cognitions may have been idiosyncratically relevant for all participants (cf. Schneider & Schulte, 2007). An aim for future studies might therefore be to redesign all stimuli such that they have a greater overall impact on and match with participants’ IBs, e.g., by targeting the subtypes often reported in PD (see Sansone & Sansone 2009). For instance, patients with PD often report symptoms concerning one particular system e.g., cardiac, respiratory, gastrointestinal, or vestibular. Identifying the bodily symptom that is most relevant and then matching the CBM-I and assessment stimuli to it might therefore increase the training’s internal validity and ultimately generalization.

Finally, our results concerning the third aim suggested that the induced variation in panic-related IBs did not affect participants’ reaction to the symptom provocation tasks. One interpretation of this result is that IBs might not be causally involved in the exacerbation or reduction of panic-related symptoms. This interpretation, however, would be in contrast with earlier findings in clinical populations where IBs have been shown to be predictive of panic disorder onset (Woud et al., 2014) and their reduction predicted symptom reduction during treatment (Teachman et al., 2010). In this light, we consider a more likely explanation to be that the variance in IBs induced by our CBM-I conditions was insufficient to have effects on panic-related symptoms in our sample. In this case we would not expect an effect of the CBM-I on symptoms (cf. Clarke et al., 2014). Notably, we were unable to shift IBs in a negative direction, and it seems plausible that we would need to induce a range of IBs from negative to benign or positive rather than only from relatively benign to positive to observe meaningful effects on panic symptom provocation tasks in an analog sample.

Another potential explanation for our null findings, also put forward by Steinman & Teachman (2010), is that the symptom provocation might not have been challenging enough to provide variance in participants’ responses, and thus may not have been an ideal panic analog. Although we aimed to increase the stressfulness of the symptom provocation tasks by increasing the number of different tasks compared to Steinman & Teachman (2010), our exploratory results showed that participants indeed reported an increase in body-related symptoms during the symptom provocation tasks, yet no increase of anxiety. Our findings could therefore indicate that in our analog sample a more impactful stressor would have been necessary for an effect of a positive versus (in our sample) relatively neutral IB to unfold. One option for further research is therefore to include symptom provocation tasks that have been shown to produce fear in healthy participants more reliably such as a vital CO2 challenge as applied by Capron et al. (2017). Another consideration regarding our stressor concerns idiosyncrasy. While we aimed to induce a variety of symptoms, it might be necessary to assess which symptoms are idiosyncratically relevant to participants to allow valenced interpretations concerning these symptoms to unfold (cf. Schneider & Schulte, 2007). As these interpretations attribute the lack of evidence for a causal role of IBs in panic to limited effectiveness of CBM-I, broader methodological considerations related to the study design that might limit effectiveness need to be taken into account. First, a one session “dose” of CBM-I may not have been sufficient to affect more distal measures of panic-related cognitive processing (for discussions on dose-response effects, see Hallion & Ruscio 2011; Jones & Sharpe, 2017). Similar mixed results on SST have occurred in other studies with single session CBM-I (e.g., Holmes et al., 2009; Yiend et al., 2014), while studies with multiple sessions of CBM-I have led to considerable change on the SST (e.g., Hirsch et al., 2020). Considering our exploratory finding that the SST but not the ERT was associated with the analog symptom measure, a generalization to the SST might be particularly important, as it has been suggested that the interpretational processing assessed via the SST is closely related to symptoms of psychopathology (Würtz et al., 2022). Accordingly, future research could investigate multiple CBM-I training sessions to increase generalization effects. However, another reason for the differential effects of CBM-I on the ERT and the SST might relate to the suggestion that interpretational processing consists of both automatic and reflective processing (e.g., Hirsch et al., 2016), and CBM-I might have a stronger effect on either of those. The questions, to which extent CBM-I targets rather automatic or reflective processing (e.g., Bowler et al., 2012) and which of these aspects is assessed via the ERT and particularly through the SST (Würtz et al., 2022) are unresolved and remain important subjects for discussion in future research. For example, applying measures of IBs that limit reflective processing in CBM-I studies, such as neurophysiological correlates (e.g., Feng et al., 2019), together with measures like the SST might shed light on both questions, which targets are aimed at more strongly via CBM-I and which processes are reflected through each measure.

Another factor that could potentially affect generalization is the context in which CBM-I is embedded. While our study investigated the effects of CBM-I as a stand-alone procedure, Capron et al. (2017) compared CBM-I in combination with psychoeducation to psychoeducation only. Their results showed that including CBM-I significantly reduced participants’ anxiety sensitivity, indicating successful generalization to another risk factor relevant to panic. Providing participants with a rationale on the relationship between IBs and symptoms in future studies or instructing them to actively apply the interpretations presented in the training in their daily life might therefore aid in improving generalization to symptoms. This approach might be useful in maximizing potential effects of CBM-I as is desired in a clinical setting to reduce symptoms. However, its application in proof-of-concept studies like the present one might be limited due to a potential increase in demand effects when participants know the rationale.

Another consideration regarding the study setup arises when comparing our study to Capron et al. (2017) and concerns the population from which our sample was drawn. While Capron et al. (2017) conducted their study with a sample with elevated anxiety sensitivity, our sample consisted of participants with a moderate level of fear of body symptoms as measured via the BSQ. Our aim in deviating from previous research that recruited based on the Anxiety Sensitivity Index was to specifically recruit participants who reported a medium level of fear of bodily symptoms instead of general anxiety sensitivity, as we considered this fear to be more specific to panic and thus our training. This was based on evidence that only anxiety sensitivity in relation to body symptoms was specifically associated with panic, while other dimensions (i.e., social or cognitive concerns) were related to broader psychopathology (Olthuis et al., 2014). However, our exploratory results that the BSQ was neither associated with the ERT nor with panic-related symptoms at pre-training question the relevance of this measure in the current study context. Additionally, as a side-effect of our recruitment process, participants’ overall anxiety sensitivity was considerably lower than in previous studies (*M* = 13.09 in our sample compared to *M* = 28.57 in Capron et al. 2017) which may have resulted in a sample less susceptible to the general set-up of this study and the CBM-I training in particular. That is, they may not have started with sufficiently negative IBs for positive training to meaningfully change reactions to the symptom provocation task, and the negative training may not have been sufficiently plausible to alter IBs in a more negative direction.

Our findings need to be interpreted in light of several limitations. First, the repeated application of symptom provocation tasks might have results in practice and habituation effects that might have overshadowed potential effects of CBM-I on the symptom provocation. Further, the fixed order of symptom provocation tasks did not allow to investigate order effects which would have been possible through a counterbalanced design. Second, our cover story that divided the training and the symptom provocation tasks into two different studies might have impeded participants in applying the interpretations from CBM-I to the body symptoms experienced during the provocation tasks. This, in turn, may have reduced generalization, and seems a sub-optimal approach when studying the effects of a training that in fact requires the active application of a newly trained cognitive style. As CBM-I in combination with psychoeducation resulted in more promising findings (Capron et al., 2017), future studies might therefore want to provide participants with a rationale for the interplay of IBs and (analog) symptoms and encourage them to actively apply those interpretations for example during symptom provocation. Third, our sample mostly consisted of healthy female participants with at least A-level education and German as a native language. Our results are thus potentially not generalizable to more diverse populations and are not translatable to patient populations. A fourth consideration concerns our control condition. Our aim was to apply a neutral control condition by applying a training with scenarios not related to the interpretation of bodily sensations. However, upon reflection, our control training may not have actually been completely *neutral*. That is, although trials were not related to (and thus neutral) with respect to panic, they were disambiguated in either a positive or negative manner, and thus not neutral in terms of valence. As discussed earlier, in our rather healthy sample, the positive endings may have been more relevant to the participants than the negative endings, rendering the control training mildly positive and thus dampening the effect size for the difference between the positive and the negative condition. Future research with the aim to investigate effects of CBM-I in comparison to a neutral control condition should therefore apply truly neutral stimuli, e.g., neutral descriptions of everyday situations as a control condition (for a discussion of adequate control conditions in the context of CBM, see Blackwell et al., 2017).

To conclude, our study provided further evidence that CBM-I can induce a positive IB concerning bodily symptoms in a healthy sample on an IB measure similar to the CBM-I training, the ERT. However, our results on the induction of a negative IB were mixed delivering no evidence as to their potential causal role in the etiology of panic. Further, due to our finding that CBM-I effects did not fully generalize to a more distal measure of IBs, the SST, and had no effects on a panic-related symptom provocation, future research on improving generalization for the purpose of experimental tests of causality, for example via adaptations of the CBM-I training and the study design, is clearly warranted.

## Data Availability

All materials (except for standardized questionnaires), including the analysis scripts and the anonymized dataset are openly available on the Open Science Framework (OSF; https://osf.io/92tfw/).
